# Relationships Between Aerobic and Anaerobic Parameters With Game Technical Performance in Elite Goalball Athletes

**DOI:** 10.3389/fphys.2018.01636

**Published:** 2018-11-20

**Authors:** Isabella dos Santos Alves, Carlos Augusto Kalva-Filho, Rodrigo Aquino, Leonardo Travitzki, Alessandro Tosim, Marcelo Papoti, Márcio Pereira Morato

**Affiliations:** ^1^School of Physical Education and Sport of Ribeirão Preto, University of São Paulo, São Paulo, Brazil; ^2^Postgraduate Program in Rehabilitation and Functional Performance, Medical School of Ribeirão Preto, University of São Paulo, Ribeirão Preto, Brazil; ^3^CIFI2D, Faculty of Sport, University of Porto, Porto, Portugal; ^4^National Brazilian Goalball Team, Department of Coaching, Jundiaí, Brazil; ^5^School of Physical Education, University of Campinas, Campinas, Brazil

**Keywords:** peak oxygen consumption, performance analysis, maximal accumulated oxygen deficit, anaerobic pathway, sport science, Paralympic sport

## Abstract

Our aims were to compare physiological parameters from the laboratory environment (LaB) and simulated goalball games (GaM), test relationships between physiological parameters in the laboratory and game technical performance (GTP), and examine the associations between physiological and technical responses during games. Seven elite athletes from the Brazilian National Team performed in LaB environment; (i) an incremental test to determine peak oxygen consumption (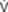
O_2PEAK_), its corresponding speed, and peak blood lactate concentration and (ii) submaximal and supramaximal efforts to estimate maximal anaerobic contribution (AnC). In GaM condition, simulated games were also performed to determine physiological responses throughout the game, and to analyze the GTP (number of throws, defenses, recovery, and density of actions). No correlations (*unclear*) were found between laboratory and games analyses for 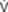
O_2PEAK_ [47.3 (17.2) vs. 25.8 (18.2) mL⋅Kg^-1^⋅min^-1^], peak blood lactate concentrations [10.2 (5.4) vs. 2.0 (0.7) mM], and total AnC [21.0 (14.0) vs. 4.8 (6.1) mL Kg^-1^]. 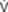
O_2PEAK_ in the laboratory condition presented *very likely* correlations with throw and recovery frequency in games (*r* = -0.87 and confidence interval [CI] = 0.41; *r* = -0.90 and CI = 0.35; respectively). Oxygen consumption remained above baseline while blood lactate concentration remained unchanged during the games. The *very likely* correlation between anaerobic alactic contribution and action density (*r* = 0.95 and CI = 0.25) highlights the importance of the alactic metabolism. In general, our study demonstrates that goalball can be characterized as a high-intensity intermittent effort, where athlete performance is based on aerobic metabolism predominance while determinant actions are supplied by the anaerobic alactic metabolism. Specifically, higher values of LaB vs. GaM highlighted the need for standardization of specific protocols for goalball evaluation, mainly for the reproduction of ecologically valid values. In addition, 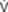
O_2PEAK_ correlated with recovery frequency in the LaB condition, demonstrating that passive or low-intensity recovery between actions is fundamental to maintain performance.

## Introduction

Goalball is a Paralympic sport for people with visual impairment. The official game comprises two halves of 12 min with three teammates on each side of the court, who successively perform offensive and defensive actions (Morato et al., 2012). The majority of studies with goalball players have examined functional, anthropometric, and morphological evaluation aspects (Çolak et al., 2004; Karakaya et al., 2009; Calıskan et al., 2011; Scherer et al., 2012; Molik et al., 2015; da Cunha Furtado et al., 2016). Other studies analyzed aerobic parameters in field tests (Çolak et al., 2004; Karakaya et al., 2009; Gulick and Malone, 2011; da Cunha Furtado et al., 2016), and technical-tactical performances in tests or during the game (Stamou et al., 2007; Bowerman et al., 2011; Morato et al., 2012, 2017, 2018; Molik et al., 2015; Link and Weber, 2018; Monezi et al., 2018). However, only one study investigated physiological responses during the game (Theophilos et al., 2005). Considering these previous studies, possible correlations between physiological fitness (e.g., indices of aerobic and anaerobic metabolisms), and physiological responses observed during the game, together with technical-tactical performances, remain unknown in the literature.

Theophilos et al. (2005) were the first researchers to analyze physiological parameters during the game. These authors reported that high-intensity physical demand (heart rates between 85–100% of the maximal) composes goalball actions, and also suggested that these high-intensity efforts are supplied by anaerobic energy, mainly using the alactic metabolism. However, to date, specific contributions of anaerobic pathways [AnAl, anaerobic lactic (AnLa), and total AnC] have not yet been investigated during the game. Presumably this is due to methodological limitations to quantify metabolic participation in this sport, which has constant contact with the floor and the ball impact during defensive actions, making the use of a continuous face mask to assess the 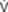
O_2_ impossible during the goalball game. In view of this limitation, previous studies used a methodological adjustment, named “the backward extrapolation technique” that can provide assessment of 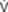
O_2,_ preserving the ecological validity. This protocol consists of coupling the gas analyzer to the athlete immediately after the effort (Leger et al., 1980; Montpetit et al., 1981; Costill et al., 1985; Campos et al., 2017a; Rodríguez et al., 2017). From the linear adjustment between 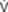
O_2_ (log transformed) and recovery time the 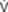
O_2_ relative to the effort is determined (Campos et al., 2017b).

Besides the use of physiological responses to verify the energy required during the game, systematic video observation can provide important information about technical and tactical demand (Hughes and Bartlett, 2002), mainly through descriptive analysis, which is proposed to identify, describe, and characterize performance (Marcelino et al., 2011). Considering the self-organizing in a goalball game (Morato et al., 2012), observational categories and performance indicators allow analysis about offensive and defensive aspects (Morato et al., 2018). The majority of recent studies have been concerned with goalball attack, mainly shot effectiveness (Link and Weber, 2018), time-motion analysis of different throwing techniques (Monezi et al., 2018), and characterization of ball time, considering the ball type and ball trajectory (Morato et al., 2018), leaving aside physiological behavior during the game and, consequently, its influence on self-organization in both offensive and defensive situations. To the best of the authors’ knowledge the relationships between physiological parameters together with game analysis have not yet been studied and could provide theoretical information to goalball coaches about game demand, mainly to prescribe specific training sessions.

Therefore, the aims of the present study were: (i) compare the relationships between physiological parameters (aerobic and anaerobic fitness), obtained in the laboratory environment and during simulated games; and (ii) investigate the relationships between physiological parameters and game technical performance. Our first hypothesis was that physiological demand throughout the game would not be correlated with the demand observed in the laboratory condition, which is mainly explained by differences in motor patterns between the situations. Considering that high-levels of physical fitness can be related to the maintenance of effort during the game, our second hypothesis was that significant correlations should be observed between physiological parameters and game technical performance.

## Materials and Methods

### Participants

Seven male elite goalball players from the Brazilian National Team participated in this study (age 20–34 years, body mass 78–98 kg, height 172–188 cm, experience level in goalball 4–22 years, and 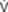
O_2PEAK_ 35.7–56.2 mL⋅kg^-1^⋅min^-1^). The athletes were categorized into three groups according to degree of visual impairment B1 (*n* = 4), B2 (*n* = 1), and B3 (*n* = 2). Their training routine is based on five sessions per week with four official games per month. The team is first position in the world ranking according to the International Blind Sports Federation (updated in April 2018), and ranked third in the Rio Paralympics Games in 2016 and in the last World Championship in 2014. All procedures were approved by the Human Research Ethics Committee of the Local University (School of Physical Education and Sport, Ribeirão Preto, Brazil; protocol number: 37889114.8.0000.5659) and were conducted according to the Helsinki declaration. Informed consent was obtained from all participants included in the study.

### Experimental Design

The Brazilian National Team was in a pre-competitive period during the evaluations. All experimental procedures (duration of 4 days) started on the 2nd day of the team’s season. It is important to highlight that the 1st day was used for the presentation of the athletes, and so they were rested. A controlled environment was built in a private room of a gym to simulate laboratory conditions (temperature: ∼21°C; relative humidity: ∼74%). Subsequently, the athletes were familiarized with the treadmill to perform a graded exercise test, after which they performed an incremental test to verify peak values of oxygen consumption, 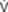
O_2_ (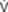
O_2PEAK_). Additionally, five randomized submaximal efforts (50–95% of 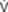
O_2PEAK_), and one supramaximal effort (110% of 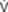
O_2PEAK_) were performed until exhaustion to determine total AnC, assumed as the maximum accumulated oxygen deficit (MAOD). These procedures were performed in this order on the 1st day and in the morning of the 2nd day of evaluations (08:00–12:00 a.m.). Thus, laboratory test parameters (LaB) were defined and aerobic/anaerobic contributions were determined. Five simulated games were played in the afternoon of the 2nd day (13:00–18:00 h) and during the whole third and fourth days for analysis of game technical performance (GTP) and physiological parameters (GaM). All tests were separated by at least 60 min of recovery and procedures were adapted considering the visual impairments presented by the players. These adaptations are described below, in specific topics.

Three dropouts occurred during the evaluations. The reasons for the dropouts were; discomfort with the mask for 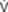
O_2_ analysis during laboratory procedures (*n* = 1), interruption of 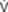
O_2_ assessment during the game (*n* = 1), and a technical fault in video recording during only one game (*n* = 1). Considering these dropouts, a sample size of five players per analysis was obtained.

### Instrumentation

Under LaB and GaM conditions, respiratory variables were determined using indirect calorimetry, which estimates energy metabolism from gas exchange analysis (Ferrannini, 1988). A gas analyzer of the Quark brand (Cosmed, Italy) was utilized, obtaining values through breath-by-breath analysis. A certified gas mixture was used to calibrate the gas analyzer. The ventilometer was calibrated every day with a 3 L syringe. These procedures were performed according to the manufacturer’s instructions. Discrepant breaths were visually removed and breath-by-breath data were used for analysis (Nieman et al., 2013). To determine [La^-^], samples (25 μL) were obtained from the ear lobe and deposited in tubes with sodium fluoride (1%). Samples were frozen at -12⋅C for further analysis (YSI-2300; Yellow Springs Instruments^®^, Ohio, United States) (Astles et al., 1996). Furthermore, the rated perceived exertion scale (RPE) was analyzed using the CR-10 (Borg, 1982), adapted by Foster et al. (2001), at the end of the incremental test. The CR-10 scale was chosen as the athletes are already familiar with this scale. For GTP analysis, simulated games were fully recorded by a single digital video camera (GoPro Hero 3+ Black Edition, Woodman Labs Inc., United States) adjusted to an acquisition frequency of 60 Hz in full HD and positioned 5 m from the center line, ensuring that all actions were recorded. The videos were analyzed using Kinovea (version 0.8.15 – motion analysis software).

### Physiological Parameters Under LaB Condition

All procedures were performed on a motorized treadmill, with some adaptations due to the visual impairment of the athletes. The procedures were conducted with sound and tactile stimuli (e.g., to show the treadmill support base). The treadmill slope was also adjusted (i.e., 8% inclination), allowing an increase in intensity, without a concomitant increase in speed.

The gas analyzer mask was presented to all athletes prior to any procedures. The incremental test started after 7 min of warm up at 5 km⋅h^-1^. The initial speed was 6 km⋅h^-1^ with increments of 1.0 km⋅h^-1^ every 120 s until voluntary exhaustion. Blood lactate concentration [La^-^] was determined at baseline and after exercise at minutes 3, 5, and 7 of recovery to analyze peak blood lactate concentration [La^-^]_PEAK_. RPE was also recorded at the end of the exercise. 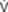
O_2PEAK_ was assumed as the highest average 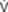
O_2_ during the final 30 s of exercise, and all athletes reached three exhaustion criteria: respiratory exchange ratio (RER) > 1.2 (0.1) a.u; [La^-^] > 9.8 (2.7) mM; and RPE = 10 (Gordon et al., 2011).

Five randomized submaximal efforts of 7 min each were completed at intensities between 50–95% of speed corresponding to 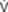
O_2PEAK_ (s
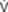
O_2PEAK_). In each effort, the mean 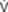
O_2_ during the final 30 s was assumed as the steady-state 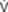
O_2_ for the corresponding velocity, and was used for the construction of the 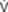
O_2_-speed relationship, which was extrapolated to estimate theoretical oxygen demand for an intensity corresponding to 110% of s
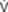
O_2PEAK_. A supramaximal effort corresponding to 110% of s
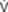
O_2PEAK_ was performed to measure the time to exhaustion, and the area below the 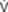
O_2_ behavior during this exercise (i.e., aerobic contribution). To ensure maximal AnC during the supramaximal effort, exhaustion should occur after 2 min of exercise (Medbo et al., 1988), which was observed for all participants. Therefore, the total AnC was calculated as the difference between total energy demand for effort (i.e., product between theoretical oxygen demand by time to exhaustion) and the area of 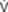
O_2_ behavior (Medbo et al., 1988). Thus, the main variables obtained in the LaB condition were AnC, 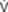
O_2PEAK,_ and [La^-^]_PEAK_.

### Physiological Parameters in GaM Condition

Prior to the simulated games, athletes performed a usual warm-up session of training (i.e., 10 min including running around the court, stretching exercises, and throw/defense actions). Subsequently, five simulated games (two halves of 12 min each, according to goalball rules) were performed and only one athlete was analyzed in each of them. During the games, five athletes played in wing position, one in center position, and one in both positions. To preserve the representativeness of the game, the pause for physiological assessment simulated a team time out request (which allows four time outs of 45 s by each team). All evaluation equipment was stored at the side of the court, in the team bench area. Seven times during the game the athletes left their position and walked toward the bench area (length ∼4 s), to be assessed: before the beginning of the game, or baseline (T0); after four (T1); eight (T2); and twelve timed minutes played of the first half (T3); followed by four (T4); eight (T5); and twelve timed minutes played of the second half (T6). To measure 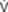
O_2_ responses, the athletes did not play with gas analyzers, and values were obtained by the backward extrapolation technique (Leger et al., 1980; Montpetit et al., 1981; Costill et al., 1985; Campos et al., 2017a; Rodríguez et al., 2017) which allows coupling of the gas equipment immediately after the effort. 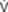
O_2_ was analyzed for at least 20 s during the physiological assessment (T0–T5), and 300 s during T6 (end of the game). [La^-^]_PEAK_ was determined at minutes 3, 5, and 7 of recovery.

The AnAl was assumed as the fastest component of post-exercise excess of 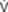
O_2_ (EPOC_FAST_), observed after T6. For this, 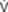
O_2_ values (log transformed) obtained during recovery were adjusted as a function of time using a bi-exponential method. The product of time constant and amplitude of the first component of this adjustment was considered as AnAl (Beneke et al., 2002; Bertuzzi et al., 2007; Zagatto et al., 2011). The AnLa was determined assuming that each unit of accumulated lactate during a game (i.e., difference between peak values observed after T6 and baseline values) is equivalent to 3 mL.O_2_ kg^-1^ of body mass (di Prampero and Ferretti, 1999; Beneke et al., 2002; Bertuzzi et al., 2007; de Campos Mello et al., 2009; Zagatto et al., 2009). The AnC was assumed as the sum of AnAl and AnLa (Bertuzzi et al., 2010). Therefore, the mean physiological variables determined in GaM conditions were 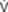
O_2_ and [La^-^] values at each evaluation during the games and AnAl, AnLa, and AnC (compared with MAOD) contributions after T6.

### Game Technical Performance (GTP)

Game technical performance is the terminology used to represent the way to perform an action to respond to a problem related with the game situation (offensive or defensive phase) (Hughes and Bartlett, 2002; O’Donoghue, 2009). To preserve the ecological validity of the game, no specific requirements were demanded of the athletes, precisely because we wanted them to play the game as if it were in a real context (“friendly” game), without influencing the situational, contextual, or positional variables, to preserve the game characteristics. All official rules were applied and only 4 interventions of 45 s (simulating team’s time out according to the rules) occurred during the game for the purposes of analysis, as explained above.

Two researchers analyzed and recorded GTP using systematic observation in specific software (Kinovea 0.8.15). Considering the six measurement moments during the game, the technical measurements comprised the following set of analysis criteria: frequency of throws, defenses, and recoveries of each athlete. The length of time (seconds) between each action (throw or defense) was considered as recovery. Density of actions (sum of throws and defenses per minute) was also calculated at each measurement moment. The technical performance indicators were selected according to both offensive and defensive principles that comprise the self-organizing in goalball (Morato et al., 2017). Reliability of GTP responses was assessed using one selected game (16.6% of the sample), analyzed twice, at different moments (15 days apart) by both observers. The Kappa index was used for categorical variables (Fleiss et al., 2013) and the ICC for numeric variables (Hopkins, 2000). For intra-observer reliability of GTP, the Kappa was 0.97 and 0.98, for actions and moments, respectively, and for inter-observer it was 1.00 for both variables. ICC ranged from 0.97 to 1.00 for intra and inter-observers at the beginning and end of recoveries.

### Statistical Analysis

Although our athletes represent an elite sample, the reduced quantity of data could influence parametric outputs. In addition to this, the Shapiro-Wilk test revealed several cases of non-normal distribution. Considering these facts, all analyses were performed using non-parametric statistics, and data are presented as median and IQR. Physiological parameters in LaB and GaM conditions were compared using Wilcoxon’s test and correlated using Spearman’s test. Physiological variables in LaB conditions were correlated to GTP using Spearman’s test. Finally, the responses observed during the seven measurement moments (T0–T6) during the games were compared using Friedman’s test, followed by Wilcoxon’s test when necessary. GaM parameters were correlated to GTP using Spearman’s test. Magnitudes of correlation coefficients were considered trivial (*r* < 0.1), small (0.1 < *r* < 0.3), moderate (0.3 < *r* < 0.5), large (0.5 < *r* < 0.7), very large (0.7 < *r* < 0.9), and nearly perfect (*r* = 1.0), according to Hopkins (2000). Spearman’s test was always accompanied by a 90% CI. Statistical Package for Social Science software, version 17.0 (SPSS Inc, Chicago, IL, United States) was used for all analyses, and the level of significance was set at *p*-value < 0.05. Furthermore, a magnitude-based inferential (MBI) statistical approach was used (Hopkins et al., 2009) (confidence level = 90%). Raw data outcomes in standardized Cohen units were utilized (ES). The quantitative chances of higher or lower differences were assessed qualitatively as follows (Hopkins et al., 2009): <1%, almost certainly not; 1-5%, very unlikely; 5-25%, unlikely; 25-75%, possibly; 75-95%, likely; 95-99%, very likely; >99%, almost certain. If the chances of higher or lower differences were >5%, the true difference was assumed as unclear. Otherwise, the effect was deemed clear (Hopkins et al., 2009). Regarding the greater impact of the present results in the field, only likely chances that the differences were true (>75%) were considered (Lacome et al., 2017).

## Results

Under laboratory conditions, the incremental test lasted 15.3 (2.4) min, the 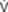
O_2_-speed relationship presented high values of linearity, *R*^2^ = 0.8 (0.1), and time to exhaustion at supramaximal effort was 2.6 (1.3) min. The results used to establish relationships between the LaB condition and simulated games are segmented into three analyses in Table 1. The first analysis compared physiological parameters (aerobic and anaerobic fitness), obtained in the laboratory (LaB) and during the simulated games (GaM). The second analysis verified the relationships between physiological parameters (aerobic and anaerobic fitness), obtained in the LaB, and GTP. The third analysis examined the relationships between GTP and GaM.

**Table 1:** Physiological and game technical parameters used in the three analyses.

	First analysis	Second analysis	Third analysis
**LaB**			
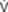 O_2PEAK_ (mL⋅Kg^-1^⋅min^-1^)	47.3 (17.2)	47.3 (14.2)	–
s 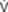 O_2PEAK_ (Km⋅h^-1^)	–	9.0 (2.3)	–
[La^-^]_PEAK_ (mM)	10.2 (5.4)	10.2 (3.5)	–
AnC (mL Kg^-1^)	21.0 (14.0)	22.2 (21.6)	–
**GaM**			
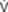 O_2PEAK_ (mL⋅Kg^-1^⋅min^-1^)	25.8 (18.2)	–	31.9 (26.1)
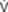 O_2MEAN_ (mL⋅Kg^-1^⋅min^-1^)	–	–	20.3 (6.3)
[La^-^]_PEAK_ (mM)	2.0 (0.7)	–	2.0 (0.4)
[La^-^]_MEAN_ (mM)	–	–	2.0 (0.9)
AnAl (mL Kg^-1^)	–	–	3.7 (4.7)
AnLa (mL Kg^-1^)	–	–	2.3 (1.6)
AnC (mL Kg^-1^)	4.8 (6.1)	–	5.8 (5.8)
**GTP**			
Throws (actions)	–	35.0 (9.0)	34.0 (26.0)
Defenses (actions)	–	67.0 (17.5)	70.0 (12.5)
Recovery (actions)	–	134.0 (21.0)	126.0 (38.5)
Density (number⋅time^-1^)	–	0.11 (0.02)	0.11 (0.03)

*LaB, physiological parameters obtained in laboratory; GaM, physiological parameters obtained during simulated game; GTP, game technical parameters; 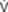
O_2_, oxygen consumption, 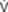
O_2PEAK_, expressed in peak oxygen consumption; 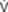
O_2MEAN_, mean oxygen consumption; s
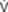
O_2PEAK_, speed correspondent to 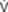
O_2PEAK_; [La^-^], blood lactate concentrations; [La^-^]_PEAK_, expressed in peak blood lactate concentrations; [La^-^]_MEAN_, mean peak blood lactate concentrations; AnC, anaerobic contribution; AnAl, anaerobic alactic; AnLa, anaerobic lactic; —-, not used during analysis; First analysis, compared the relationships between physiological parameters, obtained in LaB with physiological parameters obtained in GaM; Second analysis, tested the relationships between LaB and GTP; Third analysis, examined the relationships between GaM and GTP responses.*

**FIGURE 1:**
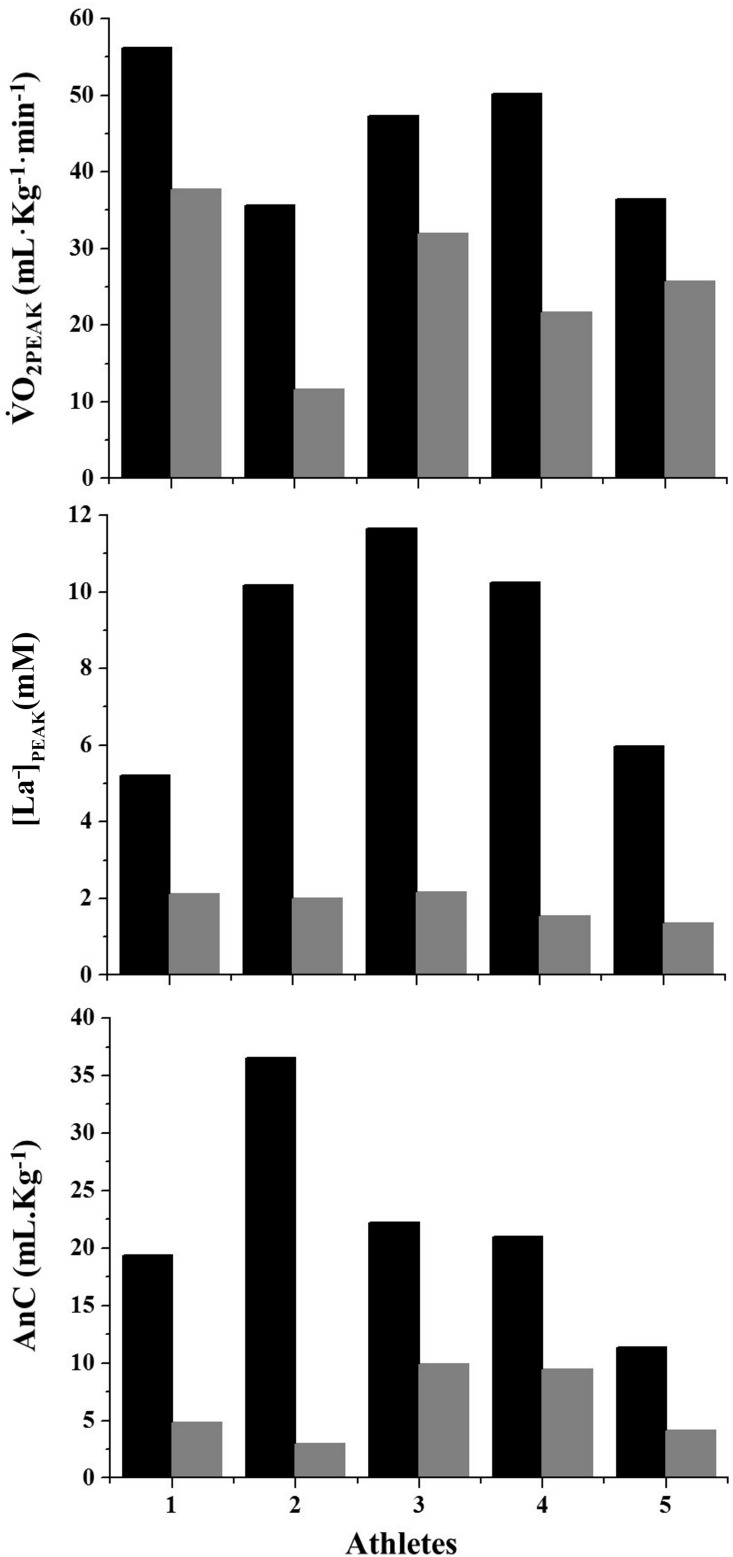
Individual values of peak oxygen consumption (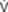
O_2PEAK_), peak blood lactate concentrations ([La^-^])_PEAK_, and anaerobic contributions (AnC), observed in the laboratory condition (black bars) and during simulated games (gray bars).

The individual values of LaB conditions and GaM variables (Figure 1) showed *unclear* correlations between evaluation methods (Table 2: *r* = 0.01–0.70; CI = 0.63–0.83). All parameters (i.e., 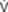
O_2PEAK_, [La^-^]_PEAK,_ and AnC) were higher in the LaB condition when compared with those of simulated games (ES = 2.46–3.34; *very likely to almost certain*), demonstrating different types of energy demand in each effort. In addition, considering the results obtained in laboratory conditions as the maximal energy capacity reached by each athlete, during the game, the energy accounted for less than sixty percent of the maximal (56, 23, and 32% of 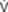
O_2PEAK_, [La^-^]_PEAK,_ and AnC, respectively).

**Table 2:** Correlations between physiological parameters determined in the laboratory condition (LaB) and simulated games (GaM).

		90% Confidence Interval	Chances analysis			
	r		+ive	Trivial	-ive	Inference
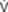 O_2PEAK_	0.70	0.63	86	5	9	Unclear
[La^-^]_PEAK_	0.30	0.83	62	10	28	Unclear
AnC	0.01	0.82	45	11	44	Unclear

*
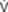
O_2PEAK_, peak oxygen consumption; [La^-^]_PEAK_, peak blood lactate concentrations; AnC, total anaerobic contribution.*

**FIGURE 2:**
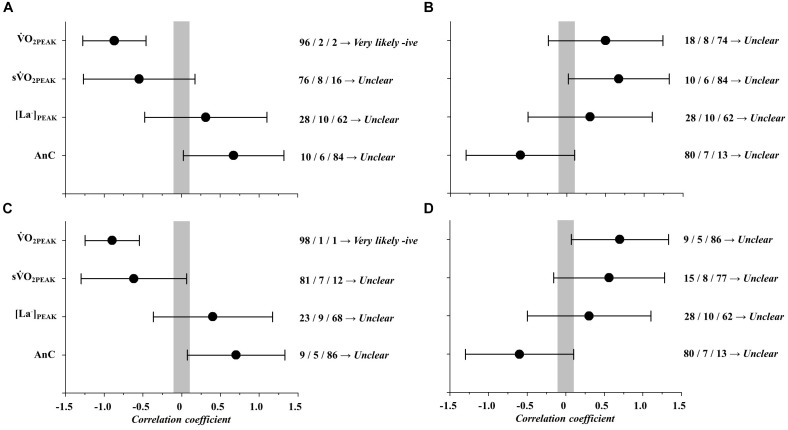
Magnitude-based inference correlations between laboratory parameters (LaB) and game technical performance (GTP). 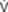
O_2PEAK_, peak oxygen consumption; s
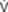
O_2PEAK_, speed correspondent to 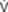
O_2PEAK_; [La^-^]_PEAK_, peak blood lactate concentrations; AnC, anaerobic contribution. **(A)** Correlation between LaB and throws; **(B)** Correlation between LaB and defenses; **(C)** Correlation between LaB and recoveries; **(D)** Correlation between LaB and density.

Correlations between LaB and GTP variables are presented in Figure 2. 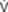
O_2PEAK_ values were *very likely* correlated to throw and recovery frequency (Figure 2A: *r* = -0.87 and CI = 0.41; Figure 2C: *r* = 274 -0.90 and CI = 0.35; respectively). No more correlations were observed between other LaB parameters (i.e., s
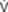
O_2PEAK_, AnC and [La^-^]_PEAK_) and GTP responses (*unclear*).

**FIGURE 3:**
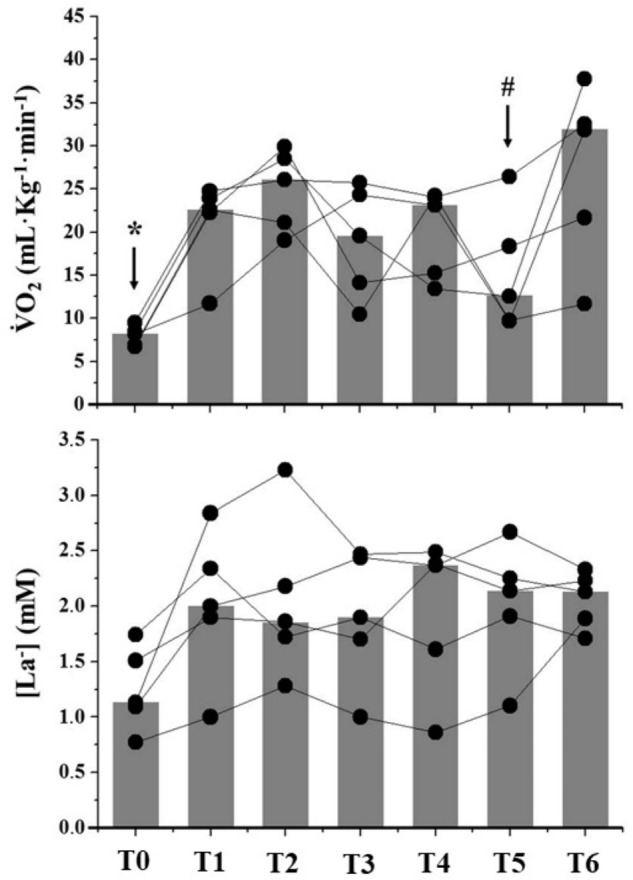
Individual responses of physiological parameters observed at the measurement moments (T0–T6) throughout the simulated game. Gray bars represent median values at each moment of assessment. ^∗^Significant differences from other measurement moments (T0 vs. T1–T6); ^#^Significant differences from T6. 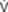
O_2_, oxygen consumption; [La^-^], blood lactate concentrations.

**FIGURE 4:**
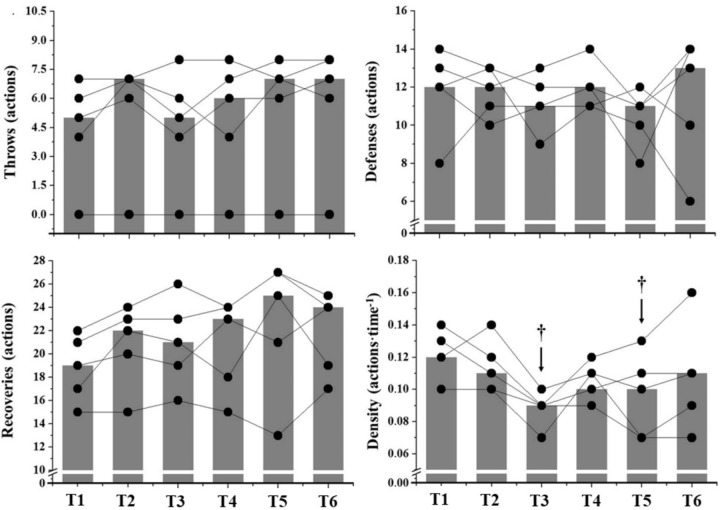
Individual responses of GTP observed at the specific measurements moments (T0–T6) throughout the simulated game. Gray bars represent median values at each assessment moment. ^†^Significant differences from T1.

**FIGURE 5:**
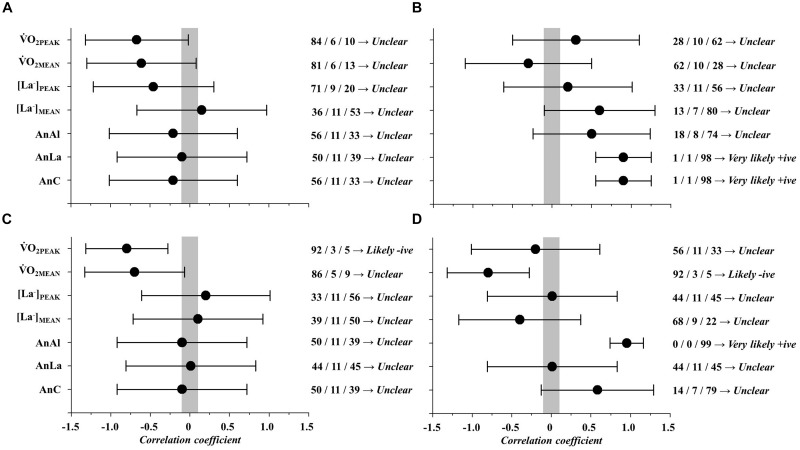
Magnitude-based inference correlations between physiological parameters during simulated games (GaM) and GTP. 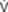
O_2PEAK_, peak oxygen consumption; 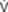
O_2MEAN_, mean oxygen consumption; s
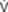
O_2PEAK_, speed correspondent to 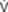
O_2PEAK_; [La^-^], blood lactate concentrations, expressed in [La^-^]_PEAK_, peak blood lactate concentrations; [La^-^]_MEAN_, mean peak blood lactate concentrations; AnAl, anaerobic alactic; AnLa, anaerobic lactic; AnC, anaerobic contribution. **(A)** Correlation between GaM and throws; **(B)** Correlation between GaM and defenses; **(C)** Correlation between GaM and recovery; **(D)** Correlation between GaM and density.

During GaM, both 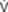
O_2_ and [La^-^] presented random responses over measurement moments (Figure 3). 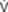
O_2_ increased from baseline during the entire simulated game, presenting highest values at T6. However, no changes in [La^-^] were observed. Random responses were observed for GTP measurements (Figure 4). One athlete did not throw during the game, which is related to his defensive role in the center player position. Figure 5 demonstrates the correlations between GaM parameters and the sum of GTP throughout the game. Both AnLa and AnC (i.e., MAOD) presented *very likely* correlations to the number of defenses during the entire simulated game (Figure 5B: *r* = 0.90 and CI = 0.35). 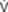
O_2PEAK_ values were *likely* correlated to recovery frequency (Figure 5C: *r* = -0.80 and CI = 0.52). Finally, 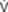
O_2MEAN_ and AnAl were *likely* to *very likely* correlated to action density (Figure 5D: *r* = -0.80 and CI = 0.52; *r* = 288 0.95 and CI = 0.25; respectively).

## Discussion

The aims of this study were to compare laboratory test parameters (LaB) and physiological variables obtained during goalball simulated games (GaM) and verify the GTP responses during the simulated games. Subsequently, relationships were verified in all conditions (LaB, GaM, and GTP). The main results were: (i) playing goalball taxes the aerobic and anaerobic alactic pathways most; (ii) higher values were observed in the LaB compared to the GaM condition, evidencing the need for standardization of specific protocols; (iii) lower recovery frequency was related to greater 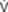
O_2PEAK_ values in the LaB condition.

Few studies have demonstrated physical responses by goalball athletes during a laboratory condition, field tests, and/or a game context. In the LaB, the majority of studies investigate the morphological profile (Çolak et al., 2004; Scherer et al., 2012), anthropometric measurements (Calıskan et al., 2011; Molik et al., 2015), and general physical fitness (Çolak et al., 2004; Karakaya et al., 2009; da Cunha Furtado et al., 2016). Gulick and Malone (2011) were the first authors to compare aerobic fitness in female goalball athletes obtained during the LaB condition (i.e., incremental test) to those observed during a field test, similar to a beep test, but performed on the goalball court. Although this attempt was important to increase the specificity of measures, the non-direct measurements of 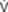
O_2_ during the field test, lack of information on its reproducibility, and lack of relationships between the real energy demand during the game, compromise the applicability of these results to practical settings, despite the strong correlation presented (*r* = 0.77). Comparing with the present study, the 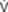
O_2PEAK_ value observed during the incremental test (34.7 ± 7.5 mL kg^-1^ min^-1^) was higher than that observed by Gulick and Malone (2011). These differences can be explained mainly by two factors: differences between participant genders (i.e., female) and the use of an ergometer (i.e., bicycle) during the incremental test.

In relation to AnC, particularly [La^-^], our result corroborates the scientific literature (Theophilos et al., 2005), demonstrating low values [3.3 (0.5 mM)] for [La^-^]_MEAN_ during goalball games. These authors argued that anaerobic lactic participation is not demonstrated during the games, and the AnAl seems to satisfy the effort demands. Considering this, our results demonstrate that physiological parameters identified in the LaB condition were higher and not correlated with GaM responses.

The relationships between LaB parameters and GTP responses demonstrated only inverse correlations between 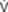
O_2PEAK_ and recovery frequency during the game. This supports the idea that higher values of aerobic fitness are related to lower recovery frequencies after high-intensity exercise (Spencer et al., 2005) contributing to maintenance of performance in subsequent actions (Glaister, 2005; Spencer et al., 2005). In addition, no correlations were observed between s
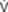
O_2PEAK_, [La^-^]_PEAK,_ and AnC to GTP responses which shows that in the LaB condition almost no physiological parameters (except 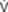
O_2PEAK_) could be related to technical performance in our sample. These results demonstrate that the use of a non-specific task to determine LaB parameters led to values not correlated with GTP responses, indicating the need for standardization of specific tests for goalball athletes, ensuring specific muscle recruitment (Peterson et al., 2016).

In our opinion, describing physiological responses in the game context is extremely important for training prescription. Correlations between physiological and technical responses are a poorly explored area in goalball, and our study could provide coaches and physiologists with a better scientific background to prescribe training sessions. In the GaM condition, 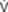
O_2_ behavior remained above baseline values during the entire game, while [La^-^] remained unchanged at all assessment moments. These results also demonstrate that the aerobic metabolism is more required during the game than the anaerobic lactic metabolism. In fact, the duration of the official game (i.e., two halves of 12 min each) indicates that the aerobic metabolism is predominant and, as discussed above, some decisive actions, such as throws and defenses, are dependent on the alactic metabolism, especially when considering their duration (i.e., 0.91 s for throws, and 1.01 s for defenses), which were similar to but lower than those evidenced by Monezi et al. (2018) (i.e., 1.82 ± 0.50). These assumptions explain the correlations between AnAl and the density of actions, indicating that this metabolism is important to perform more actions per unit of time during the game.

However, it is important to emphasize that if decisive actions are repeated without satisfactory recovery (i.e., <30 s), the contribution of the anaerobic lactic metabolism may increase. In view of the low [La^-^] observed, this situation is relatively rare during the game, and, when it occurs, the lactate is quickly removed during active recovery (Ribeiro et al., 2009). Moreover, during the game, throws are conducted by one athlete (e.g., 34 actions per game), while defensive movements are frequently performed by all athletes (e.g., 70 actions per game). As reported by Link and Weber (2018), considering all six male players during the game, the number of throws per match was nearly 185, values close to those identified in the present study. It is plausible that without satisfactory recovery these actions create lactate accumulation, explaining the significant correlations between AnLa and the number of defenses during the game. In addition, the degree of visual impairment (i.e., B1, B2, and B3) can influence performance of some actions during the game (Molik et al., 2015), as observed in the present study, where one athlete who is blinds (i.e., B1) did not perform any throws during the game.

Although our athletes represent an elite sample, the reduced quantity of data compromises some statistical analyses (e. g., type I error), which represents the main limitation of this study. Thus, our results should be interpreted with caution, especially when less experienced athletes perform the test in the LaB environment. In addition, although the validity of the backward extrapolation technique has previously been demonstrated (Leger et al., 1980; Montpetit et al., 1981; Costill et al., 1985; Campos et al., 2017a; Rodríguez et al., 2017), the game situation immediately before the interruption or before the end of the game can be influenced by the backward extrapolation technique outputs. Finally, the determination of AnAl and AnLa only after T6 could underestimate the real AnC during the game. However, considering that the athlete is only connected to the gas analyzer after exercise (during time outs), we used the backward extrapolation technique as an accessible tool to assess 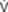
O_2_ in goalball players, enabling us to demonstrate 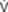
O_2_ behavior during goalball games for the first time in the scientific literature.

In general, our study demonstrates that goalball can be characterized as a high-intensity intermittent effort, where athlete performance is based on aerobic metabolism predominance, while determinant actions are supplied by anaerobic alactic metabolism. Specifically, higher values in the LaB vs. GaM highlighted the need for standardization of specific protocols for goalball evaluation, mainly for the reproduction of ecologically valid values. In addition, the correlation between 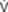
O_2PEAK_ and recovery frequency in the LaB condition show that passive or low-intensity recovery between actions is fundamental to maintain performance.

## Author Contributions

IA, CK-F, MP, and MM acquired the data, analyzed the data, interpreted the data, and wrote the manuscript. RA interpreted the data and wrote the manuscript. LT analyzed the data. AT acquired the data. All authors collaborated substantially in the development of this research, and approved the final version of the manuscript.

## Conflict of Interest Statement

The authors declare that the research was conducted in the absence of any commercial or financial relationships that could be construed as a potential conflict of interest.

## Abbreviations

[La^-^]: blood lactate concentrations
[La^-^]_PEAK_: peak blood lactate concentrations
AnAl: anaerobic alactic contribution
AnC: anaerobic contribution
AnLa: anaerobic lactic contribution
CI: confidence interval
EPOC: fast component of post-exercise excess of O_2_
ES: effect size
GaM: physiological parameters obtained during the simulated game
GTP: game technical performance
ICC: Intraclass Correlation Coefficient
IQR: Interquartile Range
LaB: physiological parameters obtained in laboratory environment
RER: respiratory exchange ratio
RPE: rating of perceived exertion
sO_2PEAK_: speed correspondent to peak oxygen consumption
O_2_: oxygen consumption
O_2PEAK_: peak oxygen consumption
